# CXC Chemokine Receptor 7 (CXCR7) Regulates CXCR4 Protein Expression and Capillary Tuft Development in Mouse Kidney

**DOI:** 10.1371/journal.pone.0042814

**Published:** 2012-08-06

**Authors:** Sammy Haege, Claudia Einer, Stefanie Thiele, Wiebke Mueller, Sandor Nietzsche, Amelie Lupp, Fabienne Mackay, Stefan Schulz, Ralf Stumm

**Affiliations:** 1 Institute of Pharmacology and Toxicology, University Hospital Jena, Friedrich Schiller University Jena, Jena, Germany; 2 Electron Microscopy Centre, University Hospital Jena, Friedrich Schiller University Jena, Jena, Germany; 3 Department of Immunology, Monash University, Central Clinical School, Alfred Medical Research and Education Precinct (AMREP), Melbourne, Victoria, Australia; Montana State University, United States of America

## Abstract

**Background:**

The CXCL12/CXCR4 axis is involved in kidney development by regulating formation of the glomerular tuft. Recently, a second CXCL12 receptor was identified and designated CXCR7. Although it is established that CXCR7 regulates heart and brain development in conjunction with CXCL12 and CXCR4, little is known about the influence of CXCR7 on CXCL12 dependent kidney development.

**Methodology/Principal Findings:**

We provided analysis of CXCR7 expression and function in the developing mouse kidney. Using in situ hybridization, we identified CXCR7 mRNA in epithelial cells including podocytes at all nephron stages up to the mature glomerulus. CXCL12 mRNA showed a striking overlap with CXCR7 mRNA in epithelial structures. In addition, CXCL12 was detected in stromal cells and the glomerular tuft. Expression of CXCR4 was complementary to that of CXCR7 as it occurred in mesenchymal cells, outgrowing ureteric buds and glomerular endothelial cells but not in podocytes. Kidney examination in CXCR7 null mice revealed ballooning of glomerular capillaries as described earlier for CXCR4 null mice. Moreover, we detected a severe reduction of CXCR4 protein but not CXCR4 mRNA within the glomerular tuft and in the condensed mesenchyme. Malformation of the glomerular tuft in CXCR7 null mice was associated with mesangial cell clumping.

**Conclusions/Significance:**

We established that there is a similar glomerular pathology in CXCR7 and CXCR4 null embryos. Based on the phenotype and the anatomical organization of the CXCL12/CXCR4/CXCR7 system in the forming glomerulus, we propose that CXCR7 fine-tunes CXCL12/CXCR4 mediated signalling between podocytes and glomerular capillaries.

## Introduction

Development of fully functioning kidney depends on coordinated crosstalk between ureteric bud tips and mesenchymal cells in the nephrogenic zone and subsequently between podocytes, endothelial and mesangial cells in the glomerulus. In detail, nephrogenesis starts with invasion of the loose metanephric mesenchyme by outgrowing ureteric buds. Contact of the two tissues induces mesenchymal condensation and ureteric bud branching by reciprocal signaling between the epithelial and mesenchymal cells. Mesenchymal condensation generates a cap mesenchyme and pretubular aggregates, which stay in contact to the ureteric bud. Perpetuated signaling from the ureteric bud then triggers mesenchymal-to-epithelial transition in pretubular aggregates and formation of polarized epithelial spheres. These renal vesicles elongate and form comma-shaped bodies which then fuse with the ureteric bud to establish tubules called S-shaped bodies [1], [2]. The proximal part of S-shaped bodies, which contains podocyte precursors, is invaded by endothelial and mesangial precursor cells. The angioblasts proliferate, assemble to create capillary loops and form functional units with both podocytes and mesangial cells [3].

Recently, the chemotactic cytokine CXCL12, an indispensable morphogen in numerous developing organs [4], [5], [6], [7], was identified in kidney stromal cells surrounding the condensed mesenchyme [8]. The CXCL12 receptor CXCR4 is described to be expressed in the condensed mesenchyme and down-regulated after mesenchymal-to-epithelial transition [8]. Although these findings suggest that changes in CXCL12/CXCR4 signaling might influence differentiation of renal mesenchyme, development of cap mesenchyme derived structures was not obviously affected in CXCR4 deficient embryos [8] – possibly because of redundancy in the chemokine system. Interestingly, mesenchymal transition of renal epithelial cells is associated with CXCL12 upregulation in a model of fibrotic kidney pathology and strong CXCR4 expression indicates progressed disease in renal carcinoma, suggesting that the CXCL12/CXCR4 pathway may be involved in epithelial dedifferentiation [9], [10]. Given that CXCL12 and CXCR4 deficient embryos exhibit severe glomerular tuft malformations, the major function of the CXCL12/CXCR4 pathway in kidney development seems related to blood vessel formation – especially glomerular vascularization [8].

Preliminary data suggest that also the atypical second CXCL12-receptor CXCR7 is expressed in the developing kidney [8]. CXCR7 modulates CXCL12/CXCR4 dependent cell migration by acting as a CXCL12 scavenger to generate local CXCL12 gradients [11], [12]. Our own data indicate that this CXCR7 decoy activity preserves the CXCL12/CXCR4 pathway in migrating neurons responding to sustained CXCL12/CXCR4 signaling by preventing CXCR4 down-regulation [11]. There exist also reports that CXCR7 induces CXCL12 dependent signaling but in contrast to most other chemokine receptors, CXCR7 fails to activate G proteins in most cell types [13], [14], [15] and CXCR7 is thought to use the G protein independent β-arrestin pathway instead [16], [17]. Anyway, CXCR7 function is essential for the development of many organs and CXCR7 deficient mice die perinatally possibly as a consequence of heart malformations [18], [19].

Here we addressed if CXCR7 is involved in kidney development. We explored patterns of CXCR7 at various embryonic stages in relation to patterns of CXCR4 and CXCL12 and examined kidneys of CXCR7 deficient embryos. Our data indicate that CXCR7 is up-regulated during mesenchymal-to-epithelial transition and that CXCR7 down-regulates CXCR4 protein and modulates CXCL12/CXCR4 dependent glomerular tuft development.

## Results

### Comparative expression pattern of CXCR7, CXCR4 and CXCL12 in the early developing kidney

**Figure 1:**
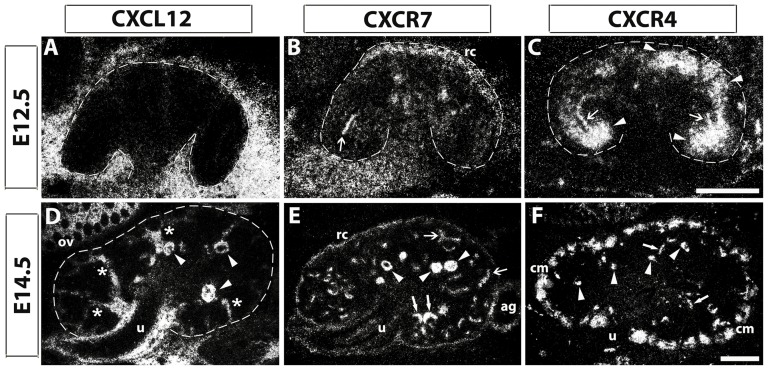
Patterns of CXCL12, CXCR7, and CXCR4 mRNAs in the early developing kidney. Adjacent kidney sections were hybridized with radiolabeled antisense riboprobes for CXCL12, CXCR7, and CXCR4 at E12.5 and E14.5. (**A**, **D**) CXCL12 expression pattern changed remarkably from E12.5 to E14.5 as no CXCL12 mRNA could be detected within the E12.5 kidney. At E14.5, CXCL12 signals are present in the kidney in stromal cells (asteristics in D), around the ureter (u) as well as in glomeruli (arrowheads in D). (**B**, **E**) CXCR7 mRNA is expressed in the region of the renal capsule (rc) and ureter at both indicated embryonic stages. The gene is also active in some ureteric buds (open arrows in B, E), immature glomeruli (closed arrows in E), and mature glomeruli (arrowheads in E). (**C**, **F**) CXCR4 mRNA is highly expressed in mesenchymal cells below the rc region at E12.5 (arrowheads in C) as well as in the cap mesenchyme at E14.5 (cm in F). CXCR4 is also expressed in some ureteric buds at E12.5 (open arrows in C). At E14.5, CXCR4 expression is found in presumptive blood vessels (closed arrows in F) and glomerular tufts (arrowheads in F). Allocation of detection signals to renal structures was performed using counterstaining with hematoxylin & eosin. ag, adrenal gland; ov, ovarium; Scale bars correspond to 100 µm.

We first investigated mRNA expression of CXCR7 to match these data with CXCL12 and CXCR4 expression patterns at embryonic stages E12.5 and E14.5 using radiolabeled in situ hybridization (Figure 1). At E12.5, CXCL12 mRNA was transiently expressed in the mesenchyme outside the developing kidney near the region of the renal capsule (Figure 1A) whereas CXCR7 was expressed predominantly in the renal mesenchyme near the forming capsule (Figure 1B: rc). This CXCR7 expression at the capsule was also detected at E14.5 (Figure 1E: rc) and persisted up to perinatal kidney (data not shown). The CXCR4 gene however was strongly expressed in more deeper layered mesenchymal cells (Figure 1C: arrowheads), which are in close proximity to outgrowing ureteric buds (UB). Some of these ureteric buds were CXCR7 and CXCR4 positive at E12.5 and E14.5 (Figure 1B,C,E: open arrows), whereas all UB tips were negative for both genes at later developmental stages (data not shown). In contrast to CXCR7 and CXCL12, CXCR4 transcripts were strongly expressed in the condensed mesenchyme at E14.5 (Figure 1F: cm) up to birth (data not shown). At this embryonic stage, CXCL12 expression was turned on in stromal cells (Figure 1D: asteristics), which derive from CXCL12 negative cells of the loose mesenchyme.

The most prominent structures though were the glomeruli. At E14.5, the glomeruli were heavily labeled for CXCR7 (Figure 1E: arrowheads) and CXCL12 (Figure 1D: arrowheads) whereas CXCR4 radiosignals were associated with the centre of the glomeruli (Figure 1F: arrowheads). In addition, CXCR7 mRNA signals were detected in pretubular structures (Figure 1E: closed arrows). Thus, CXCR7 transcripts were present in epithelial structures of diverse nephron stages. In the forming glomeruli, CXCR7 and CXCL12 genes on the one side and CXCR4 gene on the other side exhibited striking complementary activity patterns with CXCR4 expression in the glomerular center and CXCR7/CXCL12 expression in the surrounding glomerular cells (Table 1).

**Table 1:** Expression of CXCL12, CXCR7 and CXCR7 mRNA during nephrogenesis.

	CXCL12	CXCR7	CXCR4
ureteric bud tip (ep)	−	early: +	early: +
		late: (−)	late: (−)
cap mesenchyme (mes)	(+)	(+)	early: (−)
			late: +
pretubular aggregate (mes→ep)	(+)	+	+
renal vesicle (ep)	+	+	−
comma-shaped body (ep)	+	+	−
S-shaped body (ep)	+	+	−
capillary-loop glomerulus (ep)	+	+	−
mature glomerulus	+	+	+
podocytes (ep)	+	+	−
endothelial cells	−	−	+
mesangium cells	(+)	−	−

Mapping of CXCL12, CXCR7, and CXCR4 expression was performed using the markers WT1 (dual in situ hybridization) and Podocin as well as VEGFR-2 (dual immunofluorescence). +, strong expression; (+) weak expression; (−) very weak expression; − no expression. ep, epithelial cells; mes, mesenchymal cells; mes→ep, mesenchymal-to-epithelial transition.

### CXCR7 expression in epithelialized cells of the developing nephron

**Figure 2:**
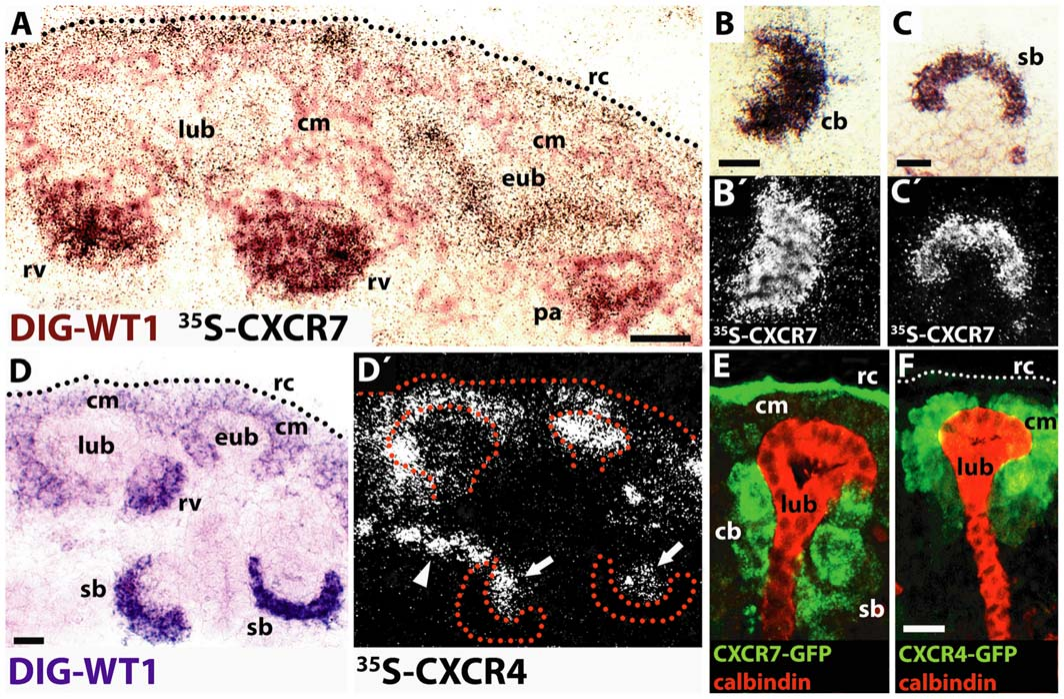
Differential expression of CXCR7 and CXCR4 in nephrogenic mesenchyme, ureteric bud, and forming glomeruli. (A–D) A digoxigenin (DIG) labeled WT1 antisense probe was used as a marker for mesenchymal and mesenchyme derived nephrogenic structures at E14.5 and co-hybridized with ^35^S labeled probes for CXCR7 or CXCR4. (**A**) Strong CXCR7 signals (black grains) are present in a T-shaped WT1 negative early ureteric bud tip (eub) which is associated with a CXCR7/WT1 co-positive pretubular aggregate (pa; black grains/brown staining). Weaker CXCR7 expression is found in a late ureteric bud tip (lub) which is associated with CXCR7/WT1 co-positive renal vesicles (rv). Note weak CXCR7 mRNA expression in the cap mesenchyme (cm) and strong CXCR7 gene activity above the cm (brown staining) at the renal capsule (rc). (**B,B′,C,C′**) Bright- and darkfield views of a comma-shaped body (cb in B) and S-shaped body (sb in C) after hybridization with a DIG labeled WT1 probe and a ^35^S labeled CXCR7 riboprobe (black grains in B,C; white grains in B′,C′). Both WT1 positive structures exhibit clear CXCR7 antisense mRNA signals. (**D,D′**) Bright- and darkfield micrographs showing WT1-positive renal tissue (D) and radiosignals of CXCR4 riboprobe (D′). Strong CXCR4 gene expression is detected in a WT1 negative early ureteric bud tip (eub in D, dotted line in D′). Weak CXCR4 labeling is seen in a late ureteric bud tip (lub) which is associated with a WT1 positive/CXCR4 negative renal vesicle (rv). Note that CXCR4 mRNA is also present in WT1 positive cap mesenchyme (cm). WT1 positive epithelial cells of S-Shaped bodies display no CXCR4 mRNA expression. The vascular cleft of S-shaped bodies (arrows in D′) as well as putative arterioles (arrowhead in D′) are CXCR4 positive. (**E**,**F**) GFP immunostaining in sections from BAC transgenic mice expressing EGFP under the control of the CXCR7 promotor (G) or CXCR4 promotor (H). Calbindin was co-stained as an ureteric bud marker. (**G**) CXCR7-GFP is highly expressed in the renal capsule (rc) as well as in comma- and S-shaped bodies (cb, sb) associated with a calbindin positive late ureteric bud (lub). CXCR7-GFP is weak expressed in cap mesenchyme (cm) and not present in the late ureteric bud. (**H**) Strong CXCR4-GFP signals are detected only in cap mesenchyme (cm). All scale bars equal 20 µm.

We then focussed on the nephrogenic zone and forming glomeruli. To distinguish ureteric buds from mesenchymal derivates, we detected transcripts of the transcription factor WT1 (Wilms' tumor protein 1), which labels mesenchyme and its derivates during the process of mesenchymal-to-epithelial transition: cap mesenchymal cells, pretubular mesenchymal aggregates as well as epithelial cells of renal vesicles, comma- and S-shaped bodies, capillary-loop nephrons, and the mature glomeruli [20]. By dual in situ hybridization with a radiolabeled probe for CXCR4 or CXCR7 mRNA (Figure 2: detected as grains), respectively, and a DIG labeled probe for WT1 mRNA (color), we identified CXCR7 and CXCR4 transcripts in WT1 negative ureteric buds. Early ureteric bud tips, which were associated with cap mesenchyme/pretubular aggregates, strongly expressed the CXCR4 and CXCR7 transcripts (Figure 2A,D′: eub). Late ureteric bud tips (Figure 2A,D′: lub), which were juxtaposed to renal vesicles, showed weaker detection signals for CXCR4 and CXCR7 antisense mRNA. CXCR4 and CXCR7 genes were expressed in WT1 positive cap mesenchymal structures as well (Figure 2A,D′: cm) however with a much more stronger expression of CXCR4 mRNA. CXCR7 labeling increased during mesenchymal-to-epithelial transition from faint at the cap mesenchyme stage to strong at the pretubular stage (Figure 2A: cm, pa). After epithelialization, CXCR7 expression remained high and was identified in renal vesicles, comma-shaped bodies, S-shaped bodies, and capillary-loop glomeruli (Figure 2A–C; Table 1). In contrast, CXCR4 labeling was faint in the ‘early’ cap mesenchyme (Figure 2D,D′: cm at eub), strong in ‘late’ cap mesenchyme (Figure 2D,D′: cm at lub) and pretubular aggregates but absent in epithelialized renal vesicles and S-shaped bodies (Figure 2D,D′: rv, sb). The vascular clefts of these S-shaped bodies, which are invaded by endothelial and mesangial cells, were heavily labeled for CXCR4 riboprobe (Figure 2D′: arrows). In addition, we detected strong CXCR4 signals in putative arterioles (Figure 1F: arrows; Figure 2D′: arrowhead).

To test these observations by an alternative approach, we examined the kidney of E16.5 BAC transgenic mice expressing EGFP under the control of the CXCR4 and CXCR7 promoters by applying dual immunofluorescent labeling for GFP and calbindin, a marker for ureteric buds. In agreement with our in situ hybridization results, CXCR7-GFP but not CXCR4-GFP was detected in the renal capsule and epithelial structures including comma- and S-shaped bodies (Figure 2E,F: rc, cb, sb). Cap mesenchyme proved strong CXCR4-GFP positive and weak CXCR7-GFP positive immunosignals whereas late ureteric buds were GFP negative in both transgenic mouse lines (Figure 2E,F: cm, lub). Similarly to CXCR7, CXCL12 mRNA was not detected in ureteric buds and the cap mesenchyme displayed only weak CXCL12 labeling (Table 1). During progression of mesenchymal-to-epithelial transition, transcription of the CXCL12 gene was turned on at the pretubular aggregate stage and CXCL12 expression remained high at subsequent nephrogenic stages (Table 1). In the vascular cleft of S-shaped bodies, hybridization signals were detected for CXCR4 (Figure 2D′: arrows) and CXCL12 (data not shown) but not for CXCR7 (Figure 2C′).

Taken together, CXCR7 and CXCR4 showed similar time dependent expression in the tip of the ureteric bud. Downregulation of CXCR4 in the ureteric bud was accompanied by CXCR4 upregulation in appendant cap mesenchyme. Whereas all three investigated mRNAs were detected in pretubular aggregates, CXCR4 expression disappeared after epithelialization. Unlike CXCR4, CXCR7 and CXCL12 were expressed in epithelial cells of the developing nephron.

### Comparative expression pattern of CXCR7, CXCR4 and CXCL12 in mature glomeruli

**Figure 3:**
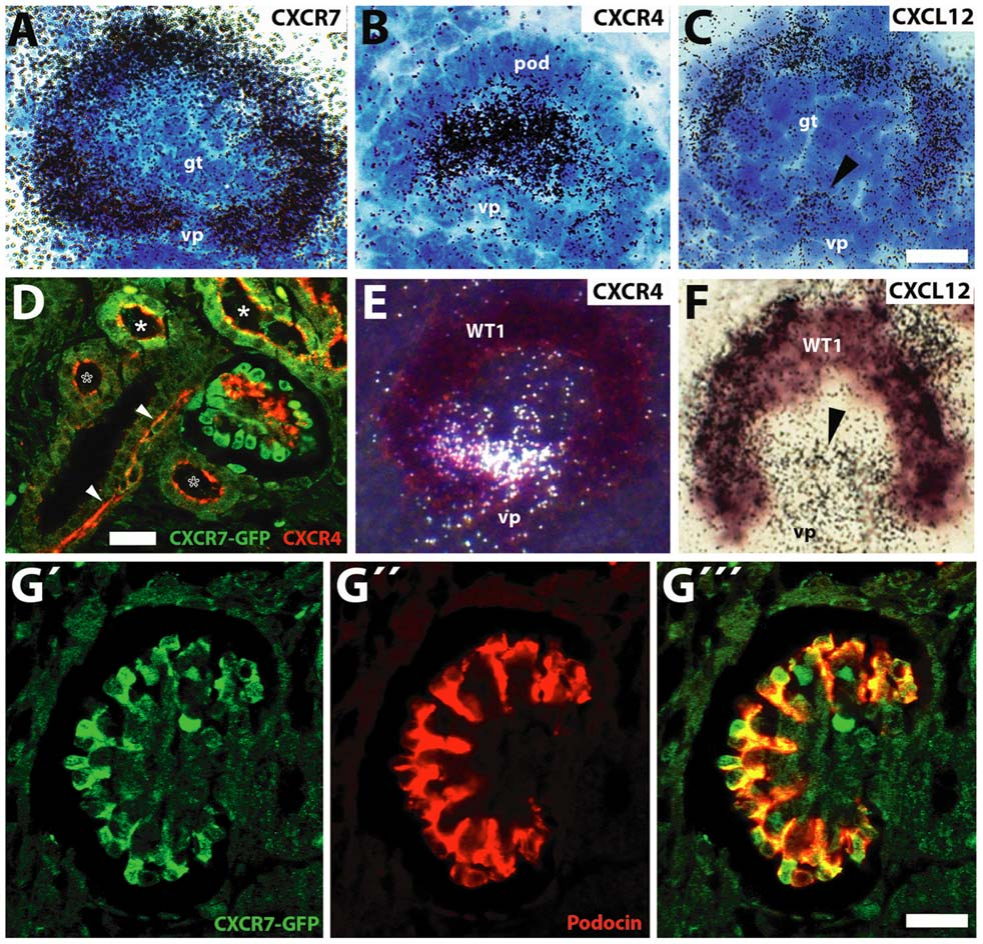
CXCR7, CXCR4, and CXCL12 expression in mature glomeruli. (A–C) Brightfield micrographs of cresyl violet stained mature glomeruli (E16.5) after hybridization with ^35^S- labeled probes for CXCR7, CXCR4, and CXCL12. (**A,B**) Signals for CXCR7 are restricted to the podocyte layer whereas CXCR4 is restricted to the center of the glomerulus. (**C**) CXCL12 is detected both in the podocytes and in the area of the vascular pole (vp, arrowhead). (D–E) Mature glomeruli after dual in situ hybridization with ^35^S labeled probes for CXCR4 (D), CXCL12 (E), and a DIG-labeled probe for podocyte marker WT1 (D,E). (**D**) The darkfield micrograph reveals CXCR4 labeling (white signals) close to the vascular pole but not in WT1 stained podocytes of the visceral blade of Bowman's capsule. (**E**) The brightfield image shows labeling for CXCL12 mRNA (black grains) in the WT1 positive podocyte layer and in the WT1 negative area of the vascular pole (vp, arrowhead). (F–G) Confocal images of dual immunofluorescent stainings for GFP/CXCR4 (F) and GFP/podocin (G) in E16.5 BAC transgenic mice expressing EGFP under the control of the CXCR7 promoter. (**F**) CXCR4 immunoreactivity is present in the glomerular tuft (arrow), presumptive arterioles (arrowheads), and at the luminar membrane of tubular epithelial cells (asteriscs). Some tubules are co-positive for CXCR7-GFP and CXCR4 (filled asteristics), others display exclusively CXCR4 protein signals (open asteristics). In the glomerulus, signals for CXCR4 and CXCR7-GFP do not overlap. (**G**) Podocytes labeled by the selective marker podocin (red) are CXCR7-GFP positive. Scale bars represent 10 µm (C,G′″) and 20 µm (F).

Consequently, we were interested to clarify the spatial relationship between CXCR7, CXCR4 and CXCL12 in mature glomeruli. Highpower analysis of hybridized sections subjected to liquid emulsion coating and counterstaining with cresyl violet revealed expression of CXCR7 and CXCL12 in the visceral epithelium of the glomerulus and that of CXCR4 and CXCL12 in the center of the glomerular tuft (Figure 3). To confirm CXCR7 expression in the visceral blade we then combined immunofluorescence for GFP and the podocyte markers podocin (Figure 3G) and nephrin (Figure S1) in E16.5 kidney sections from CXCR7-GFP transgenic mice. Examination by confocal microscopy provided evidence that the CXCR7 gene was transcribed in podocytes. Dual labeling of the CXCR4 receptor protein and CXCR7-GFP showed CXCR4 positive cells in the glomerular tuft flanking the CXCR7 expressing podocytes (Figure 3F: arrow). Absence of CXCR4 from podocytes was confirmed by dual in situ hybridization for CXCR4 mRNA and WT1 mRNA which is selectively expressed in podocytes of the mature glomerulus (Figure 3D). By applying the same approach for CXCL12, we identified CXCL12 mRNA both in WT1-positive podocytes (Figure 3E: WT1) and the WT1 negative capillary loop region (Figure 3E: arrowhead). Thus, CXCR4 and CXCR7 were strictly segregated in mature E16.5 glomeruli whereas CXCL12 was reminiscent of both CXCR4 and CXCR7.

### Histological analysis of CXCR7^−/−^ kidneys

**Figure 4:**
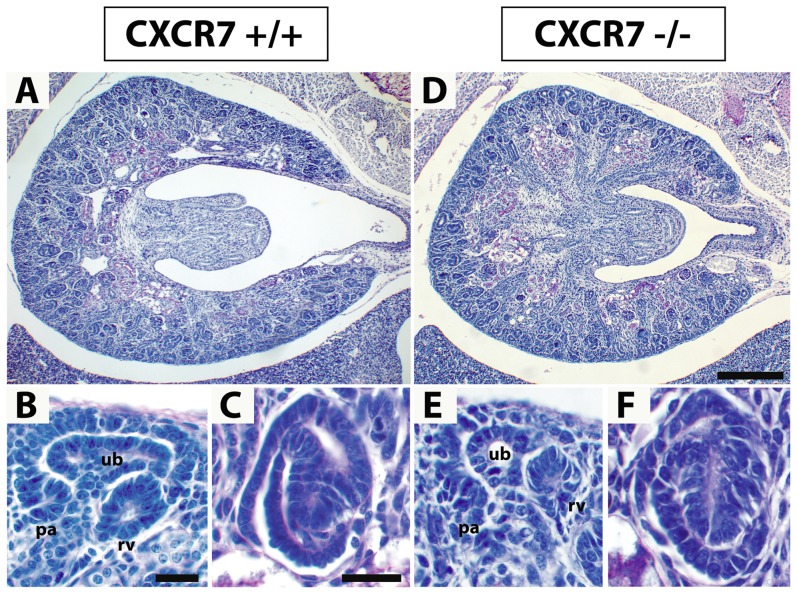
Histological analysis of CXCR7-deficient kidneys. Kidney sections of E16.5 wildtype (A–C) and CXCR7 deficient littermates (D–F) were stained with periodic acid-Schiff (PAS) reagents. (**A,D**) Macroscopic images demonstrate that the overall kidney morphology is normal in the CXCR7 knockout embryo. (**B,C,E,F**) Microscopic views of pretubular aggregates (pa), renal vesicles (rv), ureteric buds (ub) (B,E), and S-shaped bodies (C,F) do not reveal any abnormalities in the CXCR7 knockout kidney. Scale bars equal 200 µm (D) and 20 µm (B,C).

Given the strong expression of the CXCR7 gene in the developing kidney, we were then interested if kidney development was affected in CXCR7^−/−^ mice. To this end, we first analyzed counterstained serial sections from E14.5 and E16.5 CXCR7^−/−^ mice as well as CXCR7^+/−^ and CXCR7^+/+^ control littermates. Using this approach, we failed to identify any severe defects in kidney morphology and nephrogenesis (Figure 4). Both cohorts developed similar sized kidneys (E14.5: CXCR7^−/−^, 0.151±0.016 mm^3^; Control, 0.175±0.018 mm^3^) and the same number of glomeruli (E14.5: CXCR7^−/−^, 57.0±8.84; Control, 55.4±14.2). Within the glomeruli, urinary space tended to be enlarged in the CXCR7 mutants (E14.5: CXCR7^−/−^, 710.2±124.3 µm^2^; Control, 514.7±42.3 µm^2^).

### Reduced CXCR4 immunoreactivity in the cap mesenchyme of CXCR7^−/−^ mice

**Figure 5:**
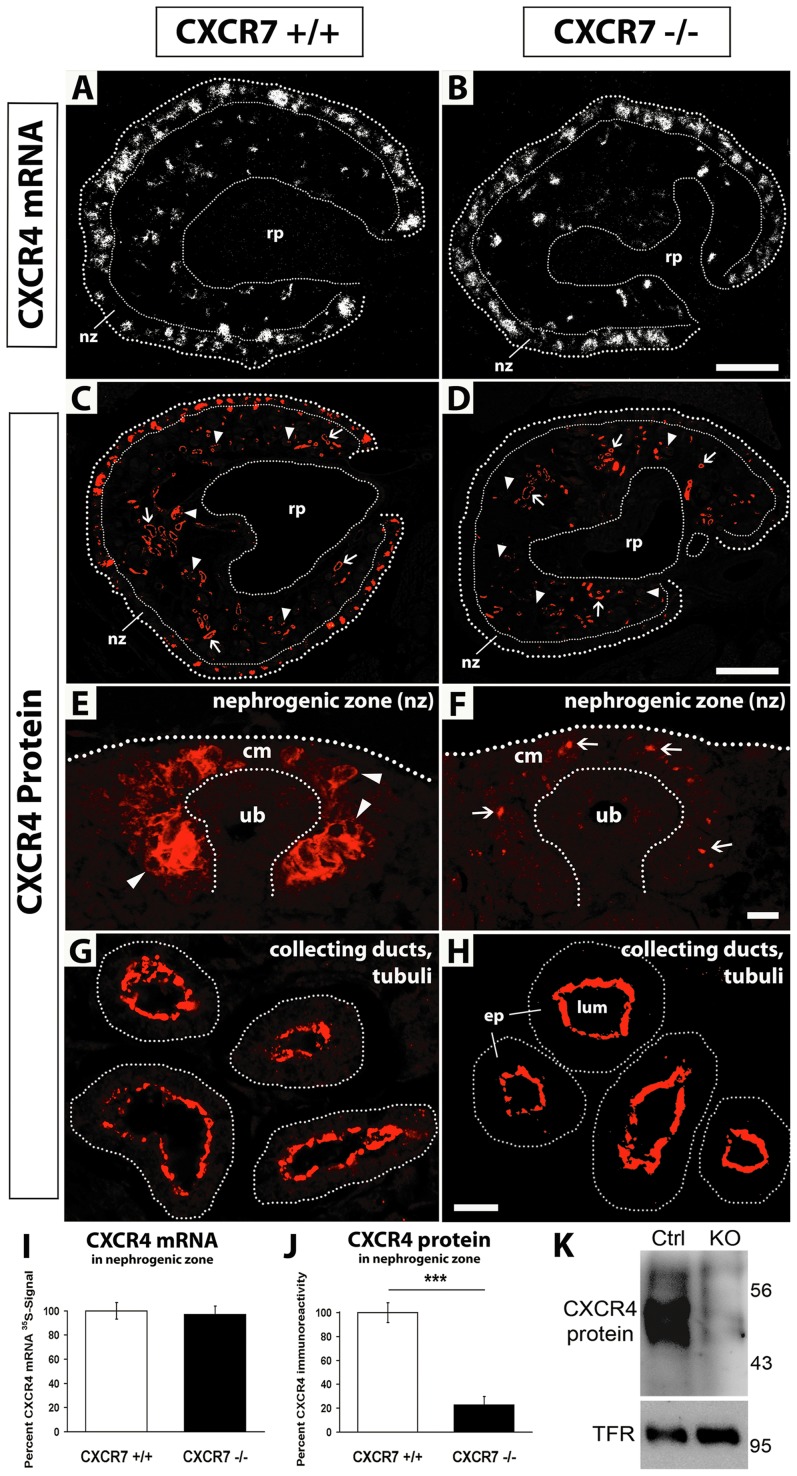
CXCR4 immunoreactivity in CXCR7 deficient kidneys. (**A,B**) In situ hybridizations with a radiolabeled CXCR4 probe show similar CXCR4 expression patterns and labeling intensities in the E14.5 kidney of a wildtype and a CXCR7 deficient mutant. (**C,D**) Immunostaining of the CXCR4 protein in E16.5 kidney sections of a wildtype and a CXCR7 mutant littermate show severely reduced immunoreactivity in the nephrogenic zone (nz), in glomerular tufts (arrowheads) but not in tubular structures (arrows) of the mutant. (**E–H**) Higher magnification pictures showing CXCR4 protein signals in wildtype and CXCR7 deficient nephrogenic zone (E,F) and tubular structures (G,H). In the cap mesenchyme (cm) of the CXCR7^−/−^ embryo (F), CXCR4 immunoreactivity is heavily reduced to single protein signals allocated to the cytoplasm of the mesenchymal cells (arrows). CXCR4 protein signals at the apical site of tubular epithelial cells (ep) are similar in a CXCR7 wildtype (G) and a mutant littermate (H). (**I**) Measurement of CXCR4 mRNA-radiosignal positive area in the nephrogenic zone (see A,B: nz). (**J**) Measurement of CXCR4 immunoreactivity positive area in the nephrogenic zone (see C,D: nz) reveals severe downregulation of the CXCR4 protein in CXCR7^−/−^ kidney sections (Mann-Whitney U test, p<0.001). Data represent mean values ±SEM as percentage of the wildtype group (I,J). (**K**) Western Blot showing CXCR4 protein expression from 22 pooled E14.5 kidneys of control and CXCR7 knockout embryos. Endogenous transferring receptor (TFR) was used as loading control. lum, lumen; rp, renal pelvis: ub, ureteric bud. Scale bars equal 100 µm (B), 200 µm (D) and 10 µm (F,H).

Because CXCR7 has been shown to influence CXCL12/CXCR4 dependent histogenesis in brain [11], [21] and heart [18], [19], [22], we then analysed the CXCR4 mRNA pattern in CXCR7^−/−^ and CXCR7^+/+^ littermates. This showed that neither the overall pattern of CXCR4 mRNA expression nor the CXCR4 mRNA positive area was changed in the CXCR7 mutants (Figure 5A,B). As we previously established that CXCR7 is required to maintain sufficient amounts of CXCR4 receptor protein to sustain CXCL12/CXCR4 dependent signaling in developing neurons [11] we examined CXCR4 immunoreactivity in the kidney of CXCR7^−/−^ embryos. Here we found that the protein signal intensity was dramatically reduced by around 75% in the nephrogenic zone (nz) as compared with control littermates (CXCR4 positive area in nz: CXCR7^+/+^, 14.08%±1.16; CXCR7^−/−^, 3.21%±0.97; p<0.001) (Figure 5C,D: nz, Figure 5J). This observation was verified by Western Blot analysis of E14.5 kidneys (Figure 5K). The CXCR4 mRNA positive area however was not altered in the nephrogenic zone of both cohorts (CXCR7^+/+^, 12.93%±0.89; CXCR7^−/−^, 12.54%±0.86) (Figure 5A,B: nz, Figure 5I), indicating that CXCR4 protein but not mRNA was down-regulated in the CXCR7 deficient cap mesenchyme (Figure 5E,F: cm). This conclusion was confirmed by the observation that CXCR4 protein signals in the wildtype could be allocated to the plasma membrane (Figure 5E: arrowheads) and those in the CXCR7 knockout to the cytoplasm of the mesenchymal cells (Figure 5F: arrows). In contrast to the nephrogenic zone, CXCR4 protein (and CXCR4 mRNA) was not diminished in the epithelium of renal tubules and collecting ducts in CXCR7 null kidneys (Figure 5C,D: arrows, Figure 5G,H).

### Perturbed glomerular tuft development in CXCR7^−/−^ mice

**Figure 6:**
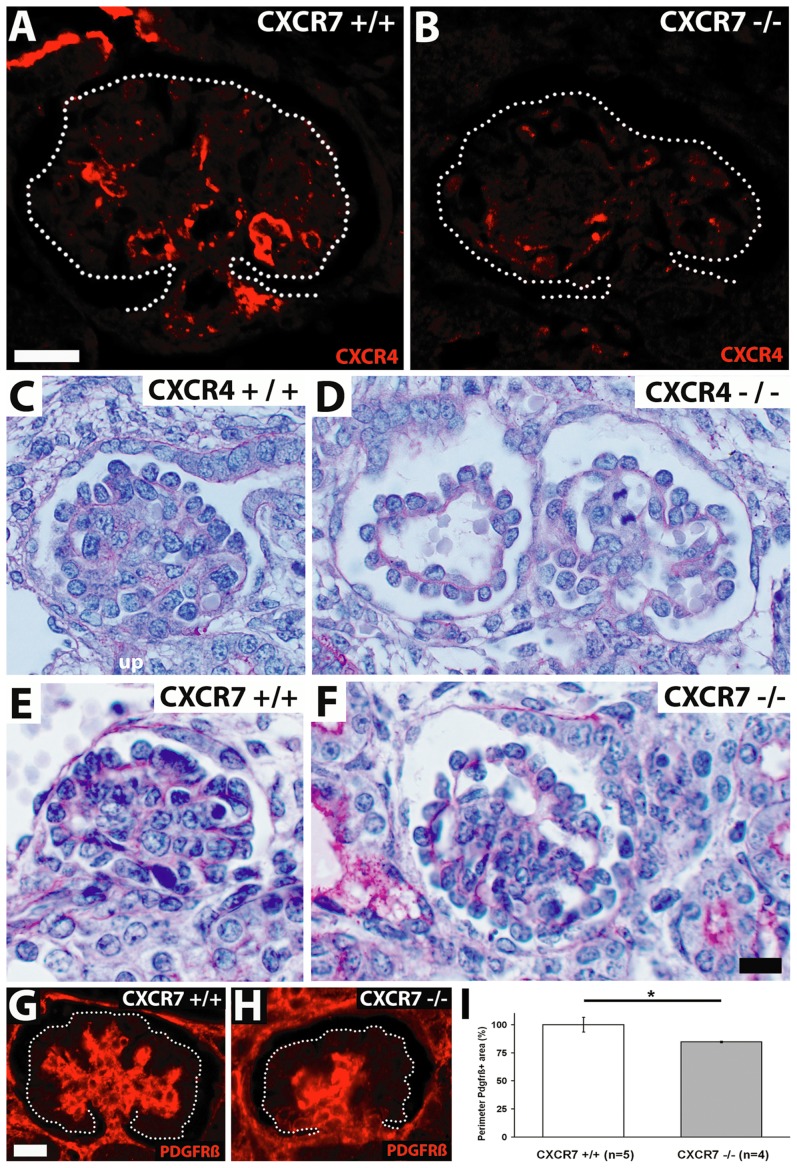
Comparison of glomerular tuft abnormalities in E16.5 CXCR7 and CXCR4 knockout mice. (**A**,**B**) Immunostainings of the CXCR4 protein in an E16.5 wildtype and a CXCR7 deficient littermate show decreased CXCR4-immunoreactivity in the glomerular tuft of the CXCR7 mutant (glomerular tufts are encircled). (**C**–**F**) PAS stained E16.5 kidney sections comparing glomeruli of wildtype (C, E) and mutant littermates (D,F). Note severe dilation of capillaries (right glomerulus in D) and a single ballooned capillary (left glomerulus in D) in the CXCR4 mutant. The glomerulus in the CXCR7 mutant (F) is similarly dilated as compared with the corresponding wildtype (E) but abnormalities within the capillary tuft are not as severe as in the CXCR4-mutant (D). (**G,H**) Micrographs of E16.5 glomeruli immunostained for the mesangium marker PDGFRβ demonstrate mesangium agglutination in a CXCR7 knockout as compared with a wildtype littermate. Dotted lines encircle glomerular tufts. (**I**) The perimeter of the mesangial tree is reduced in CXCR7 knockout mice as compared with wildtype littermates (p<0.05; Mann-Whitney U test). Data represent mean values ±SEM as percentage of the wildtype group. Scale bars represent 10 µm.

**Figure 7:**
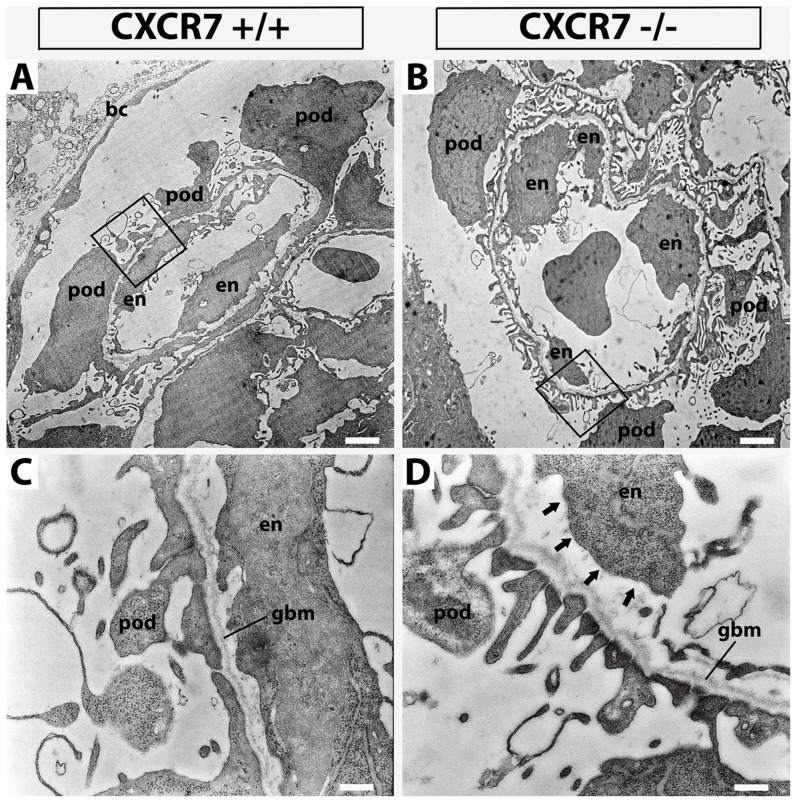
Transmission electron microscopic analysis of the glomeruli. Glomerular capillaries at 3.300× magnification (**A**,**B**) and details of the filtration barrier at 21.600× magnification (**C**,**D**; framing in A,B). CXCR7^+/+^ (C) and CXCR7^−/−^ (D) showed no differences in general morphology and glomerular basement membrane (gbm) attachment of podocytes (pod). However endothelial cells (en) seemed to be detached from the gbm (arrows). Scale bars correspond to 2 µm (A,B) and 0.3 µm (C,D).

Similar to the situation in the nephrogenic zone we found that CXCR4 immunoreactivity was diminished by around 60% in the glomerular tuft of CXCR7 mutants (Figure 5C,D: arrowheads; Figure 6A,B). Because CXCR4 is required for glomerular tuft development [8] we then focussed on a potential glomerular phenotype in CXCR7^−/−^ mice and reexamined the known phenotype in CXCR4^−/−^ mice for comparison. This showed that more than two third of all scored glomeruli contained dilated capillaries in both CXCR4 and CXCR7 mutants (Figure 6D and 6F). However, dilation of the capillaries was less severe in CXCR7^−/−^ than in CXCR4^−/−^ mice. In detail, 13.5% of all monitored CXCR7 null glomeruli displayed a severe ballooning phenotype of the glomerular capillaries (37.2% in CXCR4^−/−^) and 59.6% of the CXCR7 deficient glomeruli a mild phenotype (37.2% in CXCR4^−/−^). To check possible defects in mesangium of the CXCR7 null mutants, we performed immunostaining with the mesangial marker PDGFRβ and observed clumping of the mesangium in the mutants (Figure 6H). The perimeter of the PDGFRβ-positive area as an index for this agglutination phenotype demonstrated a clear difference between knockout and wildtype littermates (Figure 6I, p<0.05). However no alterations in total podocin and nephrin immunoreactivity were observed between the glomeruli of wildtype and CXCR7^−/−^ embryos at E16.5. This observation was confirmed by electron microscopical analysis showing no morphological abnormalities of CXCR7^−/−^ podocytes and foot processes (Figure 7C,D: pod). Endothelial cells though seemed to be detached from the glomerular basement membrane (Figure 7D: arrows).

## Discussion

Previous studies demonstrated that the CXCL12/CXCR4 pathway plays an essential role in glomerulogenesis [8], [23]. Here we reveal spatial and temporal gene expression dynamics of CXCR7, the second CXCL12 receptor, in the embryonic mouse kidney and analyses of kidney morphology in CXCR7 deficient embryos. Expression of the CXCR7 gene was up-regulated during mesenchymal-to-epithelial transition. After epithelialization and glomerular maturation, podocytes were the site of CXCR7 expression in the glomerulus. Kidneys of CXCR7 deficient embryos showed a severe reduction of CXCR4-immunoreacitivity in the nephrogenic zone and glomerular endothelium as well as malformations of the glomerular capillary loops that were reminiscent of the phenotype in CXCR4 deficient embryos.

### Mesenchymal-to-epithelial transition is associated with CXCR7 upregulation

By comparing the overall mRNA patterns of CXCR4 and CXCR7 at different stages of kidney development, it became evident that progressively more kidney structures expressed CXCR7 and progressively less expressed CXCR4. This suggests that differentiation of kidney tissues is associated with transcriptional activation of the CXCR7 and inactivation of the CXCR4 gene. At the early stage, major CXCR7 expression was indeed restricted to some ureteric bud tips and the forming organ capsule whereas CXCR4 was heavily expressed in the subcapsular mesenchyme and early ureteric bud tips. Although CXCR4 expression remained high in the subcapsular nephrogenic zone at the later stages, close examination of this region revealed that once mesenchyme had been converted into epithelial structures, CXCR4 expression was shut off. This pattern was fundamentally different from that of CXCR7 (Table 1). Before epithelialization, the nephrogenic zone was sparsely labeled for CXCR7. With the appearance of maturing nephrons though CXCR7 signals increased strongly and could be identified in podocytes and tubules. In mature nephrons, CXCR4 was detected in the glomerular capillaries and the luminar membrane of tubular cells. These patterns suggest that CXCL12 may differentially influence undifferentiated and differentiated stages of nephrogenic tissues via CXCR4 and CXCR7, respectively. The notion that strong CXCR4 expression is indicative of an undifferentiated state is consistent with a significant association of CXCR4 with advanced dedifferentiated renal cell carcinoma [10]. Interestingly, also strong CXCR7 expression was reported to be associated with poor prognosis in renal cancer [24].

Despite the remarkable plasticity of CXCR4 and CXCR7 mRNA expression in nephrogenic tissues, we observed no obvious defects in cap mesenchyme derived epithelial structures of nephrons in CXCR4 and CXCR7 deficient mice (Figure 4, Figure 7). Our findings are consistent with a previous report analyzing kidney development in CXCL12 and CXCR4 deficient embryos [8]. Thus, regulation of the CXCL12, CXCR4, and CXCR7 genes appears to be of minor importance for epithelialization in forming nephrons but might be relevant in diseases including renal carcinoma and fibrosis that are associated with epithelial-to-mesenchymal transition.

### CXCR4 protein labeling is reduced in CXCR7 deficient kidneys

As CXCR7 modulates CXCL12/CXCR4 dependent signaling [11], [12], [18], [25] we tested if the development of CXCR4 positive renal tissues requires CXCR7 by examining CXCR4 expression in the kidneys of CXCR7 deficient mice using in situ hybridization and immunohistochemistry. This showed that neither the pattern nor the level of expression of CXCR4 mRNA was altered in the absence of CXCR7. However, CXCR4 immunolabeling was strongly reduced in the nephrogenic zone (Figure 5F,J) and the glomerular tuft (Figure 6B) of the CXCR7 mutants. These findings correspond to the severely reduced level of CXCR4 protein in the telencephalon of CXCR7 deficient embryos [11]. Since CXCR7 acts as a CXCL12 scavenger and CXCR4 protein expression recovered in telencephalic explants that were kept in CXCL12 free conditions, it is likely that excess CXCL12 downregulates the CXCR4 protein in the telencephalon of CXCR7 deficient mice [11]. Our findings in the kidney suggest that a dysregulation of CXCR4 protein expression occurs not only in brain but also in non-neuronal tissues of CXCR7 knockout mice and that deletion of CXCR7 can affect CXCR4 protein expression in adjacent tissues.

### CXCR7 influences CXCL12/CXCR4 dependent glomerular vascularization

The severely reduced CXCR4 immunolabeling in the glomerular tuft of CXCR7 deficient mice prompted us to analyze glomerular vascularization in CXCR7 deficient embryos because both CXCL12 and CXCR4 are required for capillary formation in glomeruli [8]. Using CXCR4 deficient mice as reference, we identified dilated glomerular capillaries in both mutants and demonstrated that the vascular phenotype was less severe in the CXCR7 mutant (Figure 6C–F). In addition to impaired vascularization, CXCR7 deficient glomeruli showed abnormal clumps of mesangial cells and reduced branching of the mesangial tree (Figure 6G–I). A similar capillary tuft phenotype was reported previously for mouse lines lacking genes expressed by podocytes including α3β1 integrin [26], Foxc2 [27], and Pod1 [3]. The Foxc2 and Pod1 mutants exhibited a mesangial phenotype along with the vascular phenotype like the CXCR7 mutants. The fact that a loss of function in podocytes can induce a vascular phenotype indicates that podocyte-endothelial communication is essential for glomerular tuft formation. Given that CXCL12 is expressed both in mesangial cells [28] and podocytes [8], CXCR4 in endothelial cells [8], and CXCR7 in podocytes (Figure 3G), it is likely that a CXCR7 mediated CXCL12 scavenger activity influences glomerular tuft development by fine-tuning CXCL12/CXCR4 dependent communication between podocytes and endothelial cells. Moreover, a CXCL12/CXCR7 autocrine loop might be required in podocytes for development of the fully differentiated renal filtration barrier consisting of epithelialized podocytes, slit diaphragm, and endothelial cells.

### Summary and Conclusion

Up-regulation of CXCR7 gene expression is a hallmark of mesenchymal-to-epithelial differentiation in forming nephrons. CXCR7 in podocytes is strategically positioned to influence mutual CXCL12 dependent communication between endothelial cells and podocytes and, consequently, CXCR7 deficiency leads to a defect in glomerular tuft development.

## Materials and Methods

### Mice

To establish mRNA patterns of CXCR4, CXCR7, and CXCL12 in the embryonic kidney of wild type mice, pregnant C57BL/6 mice were purchased from Charles River (Sulzfeld, Germany). Embryos of transgenic CXCR7-EGFP and CXCR4-EGFP mice (The Gene Expression Nervous System Atlas project GENSAT, NINDS contract N01NS02331 to the Rockefeller University, NY, http://www.gensat.org/index.html) bred on CD1 background were used for immunohistochemistry. CXCR7^−/−^ and CXCR4^−/−^ embryos were obtained by mating heterozygous mice [17], [29] which were on C57BL/6 background. Noon of the day after mating (from 6 pm to 6 am) was defined as embryonic day (E) 0.5. Animals were housed at 40–50% relative humidity and 21±1°C (12/12 hour dark/light cycle) with food and water ad libitum. All animal procedures were in accordance with German and EU guidelines and approved by the Thüringer Landesamt für Lebensmittelsicherheit und Verbraucherschutz (Reg.-Nr. 22-2684-04-02-015/11), Tennstedter Straße 8/9, 99947 Bad Langensalza.

### Riboprobes and in situ Hybridization procedures

The CXCL12 probe was transcribed from full-length mouse SDF-1alpha cDNA [30]. Probes for mouse CXCR4 and mouse CXCR7 corresponded to the receptors' coding regions [11], [31]. A cDNA used to generate a mouse WT1 probe was kindly provided by C. Englert (Leibniz Institute for Age Research-Fritz Lipmann Institute Jena, Germany) [32]. All described cDNAs were subjected to DNA sequencing for control. Riboprobes were generated from the linearized vector constructs by in vitro transcription using [^35^S]-UTP and [^35^S]-CTP (1250 Ci/mmol; PerkinElmer, Rodgau, Germany) or digoxigenin-UTP (DIG; Roche, Mannheim, Germany) as label. The probes were subjected to mild alkaline hydrolysis and purified using P-30 spin columns (Bio-Rad, München, Germany). For hybridization, riboprobes were diluted in hybridization buffer (0,5 M NaCl, 1 mM EDTA, 10 mM Tris-Cl, 20 mm dithiothreitol, 1× Denhardt's solution, 20 mg/ml yeast tRNA, 10% dextran sulfate, and 50% formamide) to yield concentrations of 50,000 cpm/µl. Hybridization, washing, and detection of radioactive probes were performed as described [33]. For highpower bright- and dark-field microscopic analysis with the AXIO Imager.A1 microscope (Carl Zeiss, Jena, Germany), autoradiographic ^35^S-signals were detected with NTB nuclear emulsion (Eastman Kodak, Rochester, NY) after 21 d exposure. Cresyl violet was used as a counterstain. For detection of two different RNA transcripts in the same tissue section, ^35^S and DIG labeled probes were co-hybridized after adding DIG riboprobes to the appropriate radioactive hybridization solution at a final concentration of 1 µg/ml. Hybridization, washing, and detection of DIG labeled probes were carried out as described [31]. For detection of the co-hybridized ^35^S probes, slides were covered with 66% K.5 emulsion (Ilford, Mobberley, UK) diluted in water. Exposure times were 21 d.

### Histochemistry, Antibodies, and Immunohistochemistry

Mouse embryos were fixed in Bouin/Hollande fixative for 16 hrs (E14.5) and 24 hrs (E16.5) and subjected to routine paraffin embedding [34]. Sections were cut at 8 µm for immunohistochemistry and 4 µm for histochemical staining with Hematoxylin/Eosin or Periodic acid-Schiff (PAS). Immunolabeling was performed as described [35]. Briefly, endogenous peroxidase was blocked with methanol/H_2_O_2_ after deparaffinization. For antigen retrieval, sections were bathed for 25 min in 0.01 M citrate buffer (pH 6.0) at 95°C. Sections were then blocked in 50 mM phosphate buffered saline, pH 7.4 (PBS) containing 5% bovine serum albumin (BSA), and incubated overnight with primary antibody in 50 mM PBS containing 1% BSA. The following primary antibodies were used: UMB-2 rabbit monoclonal anti-CXCR4 [36] (Figure S2), rabbit anti-GFP (#132002, Synaptic Systems, Göttingen, Germany), goat anti-GFP (ab5450, Abcam, Cambridge, UK), rabbit anti-Calbindin (CB-38a, Swant, Marly, Switzerland), guinea-pig anti-nephrin (GP-N2, Progen, Heidelberg, Germany), rabbit anti-podocin (P-0372; Sigma-Aldrich, Taufkirchen, Germany), and rabbit anti-PDGFRβ (28E1, New England Biolabs, Frankfurt, Germany). Biotinylated and fluorescent donkey secondary antibodies (Dianova, Hamburg, Germany) were used at 1∶200. For dual fluorescence, primary antibodies from different species were combined. TSA enhancement (Vector ABC kit; Vector Laboratories, Lörrach, Germany) was performed prior to detection with AF555 and AF488 (Invitrogen, Karlsruhe, Germany) as described [37] for rabbit anti-GFP, rabbit anti-Calbindin, guinea pig anti-nephrin, and rabbit anti-podocin.

### Analysis of CXCR4 Expression by Western Blot

For detection of CXCR4 protein in kidneys of E14.5 mice, kidney samples from 11 CXCR7 knockout and 11 control littermates were pooled in 1 ml RIPA buffer, sonicated for 5 s, and gently inverted for 1 hr at 4°C before centrifugation for 30 min at 23,000× g at 4°C. Glycoproteins were enriched using wheat germ lectin agarose beads as described [31]. Because the UMB-2 antibody used for detection of CXCR4 [36] recognizes the non-phosphorylated CXCR4 [11], samples were dephosphorylated by incubating the beads for 3 hr at 37°C in 170 µl 1× NEBuffer for Protein Metallo Phosphatases supplemented with 400 units lambda protein phosphatase (#P0753, New England Biolabs, Frankfurt, Germany). Beads were washed with RIPA buffer before and after dephosphorylation. Proteins were eluted for 15 min at 60°C with SDS sample buffer. Samples were then subjected to 10% SDS-polyacrylamide gel electrophoresis and immunoblotted onto nitrocellulose. To demonstrate equal glycoproteins loading, endogenous transferrin receptor was detected using monoclonal mouse anti-transferrin receptor antibody (136800, Invitrogen, Karlsruhe, Germany).

### Quantitative analyses

Analysis of in situ hybridization with CXCR4 riboprobes was performed with each six kidney sections of E14.5 CXCR7^−/−^ (n = 3) and CXCR7^+/+^ (n = 3) littermates (see Figure 5A,B).

Immunostainings of podocin, nephrin, CXCR4, and PDGFRβ were quantitatively analyzed in E16.5 CXCR7 knockout and wildtype littermates. In each CXCR7^−/−^ (n = 4) and CXCR7^+/+^ (n = 5), two kidney sections per animal (see Figure 5) and five randomly selected mature glomeruli per animal (see Figure 6), respectively, were photographed. Using the manual drawing tool of the ImageJ software, the area of the nephrogenic zone (see Figure 5) and the glomerular tuft (see Figure 6) was defined as region of interest and measured. The immunostained area was then determined after setting a uniform threshold level and expressed as percentage of the area of interest. A mean value was calculated for each animal. The CXCR7 knockout and control cohorts were compared using a Mann-Whitney U test.

Kidney size was determined for eight E14.5 CXCR7^−/−^ and nine control littermates by measuring the area of the organ in 50 µm intervals in Hematoxylin/Eosin stained horizontal sections serially cut through the embryo. Area measurements were integrated to calculate the kidney volume. Number of glomeruli after capillary loop stage was determined using the same set of sections.

Ballooning phenotype was monitored in a blind manner on PAS stained 4 µm kidney slices of eight CXCR7^−/−^ and nine CXCR7^+/+^ embryos at E16.5 (CXCR4^−/−^ and CXCR4^+/+^, each n = 2) using a score that distinguished severe, mild, and no dilation of glomerular capillaries.

### Transmission electron microscopy

The tissue was fixed with a combination of formaldehyde (4%) and glutaraldehyde (2%) in cacodylate buffer (0.1 M, pH 7.4) over night at 4°C. After washing tree times with cacodylate buffer a post fixation with 1% osmium tetroxide in cacodylate buffer was done. Within the following ascending ethanol series staining with 2% uranyl acetate was performed. After embedding in araldite resin 80 nm ultra thin sections were cut and finally stained with lead citrate. The samples were investigated using a EM 900 (Zeiss, Oberkochen, Germany) transmission electron microscope.

## Supporting Information

Figure S1 **Expression of CXCR7-GFP in podocytes.** Dual immunofluorescence for GFP and the podocyte marker nephrin in a kidney section of a E16.5 CXCR7-GFP BAC transgenic mouse demonstrates colocalization of GFP and nephrin. Scale bar equals 20 µm.
(TIF)

Figure S2 **Specific CXCR4-labeling by the UMB-2 anti-CXCR4 antibody.** UMB-2 antibody was applied to kidney sections from E16.5 wildtype and CXCR4 knockout mice and detected by the 3-amino-9-ethyl-carbazole method. Sections were counterstained by Hematoxylin & Eosin. UMB-2 specifically detects CXCR4 protein in the cap mesenchyme (cm), in a presumptive blood vessel (arrow), in cells connecting S-shaped bodies (open arrowheads) and within the tuft of mature glomeruli (closed arrowheads). Scale bar equals 100 µm.
(TIF)
