# Supplementary figures and images for: CXC Chemokine Receptor 7 (CXCR7) Regulates CXCR4 Protein Expression and Capillary Tuft Development in Mouse Kidney
Source: PLoS One. 2012 Aug 6;7(8):e42814. doi: 10.1371/journal.pone.0042814 (PMC3412803; doi:10.1371/journal.pone.0042814)

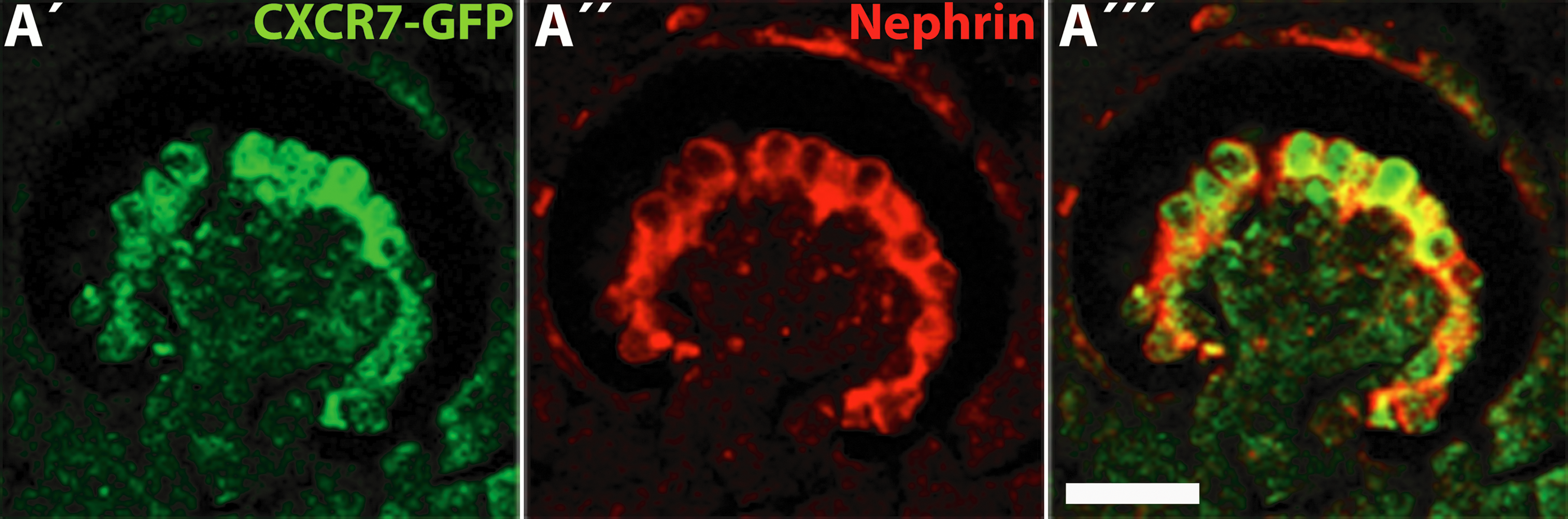

Supplement: Figure S1 — Expression of CXCR7-GFP in podocytes. Dual immunofluorescence for GFP and the podocyte marker nephrin in a kidney section of a E16.5 CXCR7-GFP BAC transgenic mouse demonstrates colocalization of GFP and nephrin. Scale bar equals 20 µm. (TIF) [file pone.0042814.s001.tif]

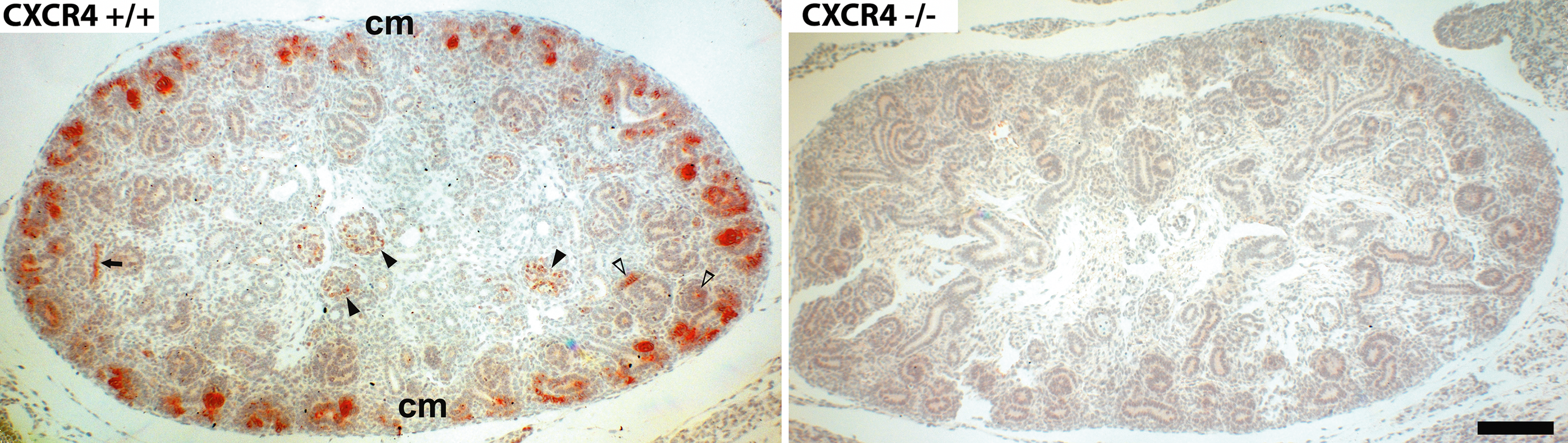

Supplement: Figure S2 — Specific CXCR4-labeling by the UMB-2 anti-CXCR4 antibody. UMB-2 antibody was applied to kidney sections from E16.5 wildtype and CXCR4 knockout mice and detected by the 3-amino-9-ethyl-carbazole method. Sections were counterstained by Hematoxylin & Eosin. UMB-2 specifically detects CXCR4 protein in the cap mesenchyme (cm), in a presumptive blood vessel (arrow), in cells connecting S-shaped bodies (open arrowheads) and within the tuft of mature glomeruli (closed arrowheads). Scale bar equals 100 µm. (TIF) [file pone.0042814.s002.tif]
